# Objective stratification of knee osteoarthritis stages using a semi-supervised learning approach on multimodal MRI-CT cartilage features

**DOI:** 10.3389/fdgth.2026.1824659

**Published:** 2026-06-25

**Authors:** Federica Kiyomi Ciliberti, Ida Maruotto, Halldor Jonsson, Paolo Gargiulo

**Affiliations:** 1 Institute of Biomedical and Neural Engineering, Reykjavik University, Reykjavík, Iceland; 2 Department of Science, Landspitali, University Hospital of Iceland, Reykjavik, Iceland

**Keywords:** cartilage morphology, cartilage radiodensity, CT, disease stratification, imaging biomakers, knee osteoarthritis (KOA), multimodal imaging, semi-supervised learning

## Abstract

**Introduction:**

Knee osteoarthritis (KOA) is a chronic and progressive joint disease that affects middle-aged and older adults. Early detection is crucial to prevent progression toward joint replacement and improve long-term outcomes, yet current diagnoses are strongly influenced by subjective symptoms, especially pain perception, which varies widely across individuals and does not reliably reflect structural degeneration. This study introduces a semi-supervised learning (SSL) framework for characterizing KOA stages through combined MRI and CT-derived cartilage features.

**Methods:**

A cohort of 133 knee scans was analyzed, including 36 expert-labeled cases categorized as healthy, early degeneration, or advanced degeneration. These labels served as seeds for graph-based SSL using Label Propagation and Label Spreading, producing pseudo-labels for the remaining samples.

**Results:**

Label stability across ten Monte Carlo runs demonstrated high agreement (0.91 ± 0.14) and substantial reliability (Fleiss’ kappa = 0.781). Supervised classifiers trained on the SSL-labeled dataset achieved robust performance, with Support Vector Machines and Logistic Regression yielding the highest weighted F1-scores (0.84 and 0.81, respectively). Statistical analysis confirmed significant differences among the three classes for all extracted features.

**Discussion:**

The volume-to-surface ratio and density heterogeneity demonstrated the strongest discriminatory power, reflecting progressive cartilage thinning, surface irregularity, and increasing structural heterogeneity consistent with KOA pathophysiology. These results show that combining expert knowledge with SSL enables reliable KOA stratification even with limited labeled data, offering meaningful insights into cartilage degeneration and laying the foundation for quantitative and more objective imaging-based biomarkers and future continuous scoring systems.

## 1 Introduction

Knee osteoarthritis (KOA) is a chronic and progressive joint disease that affects middle-aged and older adults and is the most prevalent form of osteoarthritis worldwide (1). It is characterized by the gradual breakdown of articular cartilage between bone ends, together with inflammation and changes in the surrounding bone and soft tissues, leading to joint pain, stiffness, and deformity. In severe cases, KOA can be one of the primary causes of disability (2, 3). Early KOA detection is crucial to prevent progression toward joint replacement surgery (4), yet diagnosis remains challenging due to the disease’s complex and multifactorial nature. Clinical assessment typically combines imaging and patient-reported symptoms such as pain and functional limitation. However, these subjective indicators are not always reliable proxies of disease severity or progression. Pain perception, in particular, varies substantially across individuals, which can lead to misclassification, especially at early stages of degeneration. Regarding imaging, X-ray remains the first-line modality due to its affordability and ability to detect classical KOA signs such as joint space narrowing, osteophytes, and subchondral sclerosis (5). Accordingly, the current gold standards for KOA diagnosis are the Ahlbäck and Kellgren–Lawrence classification systems, which are based on radiographic features such as joint space narrowing, osteophyte formation, and bone deformity (6). Nevertheless, subjectivity plays a pivotal role in these grading systems, as interpretations depend on observer experience. While substantial inter-observer agreement has been reported, reliability improves with clinical expertise, and intra-observer consistency is lower among less experienced clinicians (7). Moreover, X-ray imaging is limited in detecting early cartilage damage and soft tissue changes. Magnetic resonance imaging (MRI) offers a more comprehensive evaluation by visualizing cartilage, menisci, bone marrow alterations, and synovial tissue, and is particularly valuable for identifying early or subtle changes not visible on X-ray, albeit at higher cost. Computed tomography (CT) and hybrid techniques such as PET-MRI are less commonly used but can provide complementary information in selected cases (5). In particular, CT has been employed to study KOA-related joint alterations such as trabecular bone remodeling, development of subchondral cysts, and bone sclerosis (8).

In musculoskeletal diseases, timely detection supports early intervention, reduces irreversible damage, and improves long-term outcomes. Advances in imaging, biomarkers, and artificial intelligence (AI) are transforming the early identification of osteoarthritis and other musculoskeletal disorders (9, 10). Although no current therapy can modify the course of KOA, early detection remains essential to guide future disease-modifying strategies and improve patient management.

Machine learning (ML) has increasingly been adopted to support diagnosis by enabling earlier detection than traditional approaches, improving diagnostic accuracy, and reducing costs across a range of conditions. Most ML methods rely on supervised learning, which requires large amounts of accurately labeled data. In medical contexts, however, comprehensive and reliable annotations are often difficult to obtain due to the need for expert knowledge, time constraints, and inherent subjectivity. As a result, alternative paradigms such as unsupervised learning, which requires no labeled data, and semi-supervised learning (SSL), which leverages only a small set of labeled samples alongside a larger unlabeled dataset, have gained increasing attention. SSL is particularly well suited for medical imaging applications, where acquiring high-quality ground truth labels is costly and often impractical, yet large volumes of unlabeled data are routinely available. In this context, recent studies have proposed SSL frameworks for early KOA detection using X-ray images, including self-ensembling teacher–student networks (11, 12), semi-supervised anomaly detection (13), and active learning with consistency regularization (14). Recent studies have proposed SSL frameworks for early KOA detection using X-ray images, including self-ensembling teacher–student networks (11, 12), semi-supervised anomaly detection (13), and active learning with consistency regularization (14). These approaches demonstrate that SSL can achieve performance comparable to, or better than, fully supervised methods while requiring fewer labeled samples (12, 14). Deep learning architectures such as ResNet, EfficientNet, and InceptionV3 have also been widely used for KOA classification on MRI and X-ray data, achieving strong performance in moderate and severe disease stages (15–17). Ensemble and hybrid models have further improved accuracy, in some cases exceeding 99% for KOA detection (15, 16). However, most existing works rely on X-ray or MRI alone, with very few incorporating CT data. While CT effectively captures density-related properties and bony changes, it is rarely used for cartilage assessment, which remains the primary tissue affected by KOA.

Despite advances in imaging and computational analysis, current diagnostic pathways remain strongly influenced by subjective elements, most notably pain. Pain intensity does not reliably reflect the biological state of the joint: individuals with similar structural degeneration may report markedly different symptoms, while significant discomfort may occur despite limited morphological change. Consequently, clinical decisions can reflect perceived symptom severity rather than objective tissue degeneration, introducing structural bias and limiting early detection. These issues are compounded by the lack of reliable ground truth labels, as radiological assessments are affected by observer variability and inconsistent correlation with true cartilage degeneration.

To overcome these limitations, there is a need for quantitative, imaging-derived metrics that capture cartilage degeneration independently of symptoms, enabling objective assessment free from pain-driven bias. In this context, we structured our analysis around three imaging-based classes—healthy, early degeneration, and late degeneration—to represent the structural progression of KOA while decoupling it from clinical symptom variability. In real-world clinical environments, complete and consistently annotated labels, are rarely available, limiting the applicability of fully supervised ML approaches (18). By integrating MRI-based segmentation with CT-derived density and morphological measures under a semi-supervised framework, it becomes possible to leverage multimodal information despite incomplete labeling, enabling an objective and interpretable characterization of knee cartilage degeneration.

## 2 Materials and methods

### 2.1 Cohort and knee scans

A total of 93 volunteers of varying ages were recruited as part of two EU research projects focused on developing new solutions for cartilage regeneration and KOA: the RESTORE project (https://restoreproject.eu/) and the SINPAIN project (https://www.osteoarthritis-sinpain.eu). Approval for the study was obtained from the Icelandic Bioethics Commission (approval number: VSN-19-050V1, approval date: 25/10/2022). All participants provided informed consent.

The study included individuals experiencing knee pain at various stages of KOA as well as volunteers who had no current pain or cartilage degeneration and no history of trauma. Each participant underwent both MRI and CT scans of the knee during the same visit, ensuring consistent acquisition conditions. Some volunteers had both knees scanned, resulting in a total of 133 knee datasets. Because volunteers were not preselected based on clinical history, the presence or absence of pain did not necessarily reflect the underlying structural condition of the joint. Pain perception varies considerably across individuals: some may experience significant discomfort even with mild degeneration, while others may remain asymptomatic despite advanced structural changes. As a result, both symptomatic and asymptomatic participants potentially presented a range of degeneration stages, including subclinical cases that would not have been identifiable based solely on symptoms.

The scans presented the following configurations:

- CT: A 320-slice Toshiba Aquilion One scanner acquired 0.5 mm slices (0.25 mm increment) over a 15–16 cm knee-centered region at 120 kV and 250 mA without contrast, with patient-specific dose modulation based on a reference CTDI of 12.1 mGy and DLP of 193.2 mGy cm.
- MRI: A 3T Siemens Prisma scanner acquired 3D isotropic 0.6 mm fat-suppressed intermediate-weighted fast spin echo sequences using a surface coil over a 14–16 cm knee-centered field of view, enabling high-resolution multiplanar assessment of cartilage and subchondral bone.

### 2.2 Image processing and feature extraction

**Figure 1:**
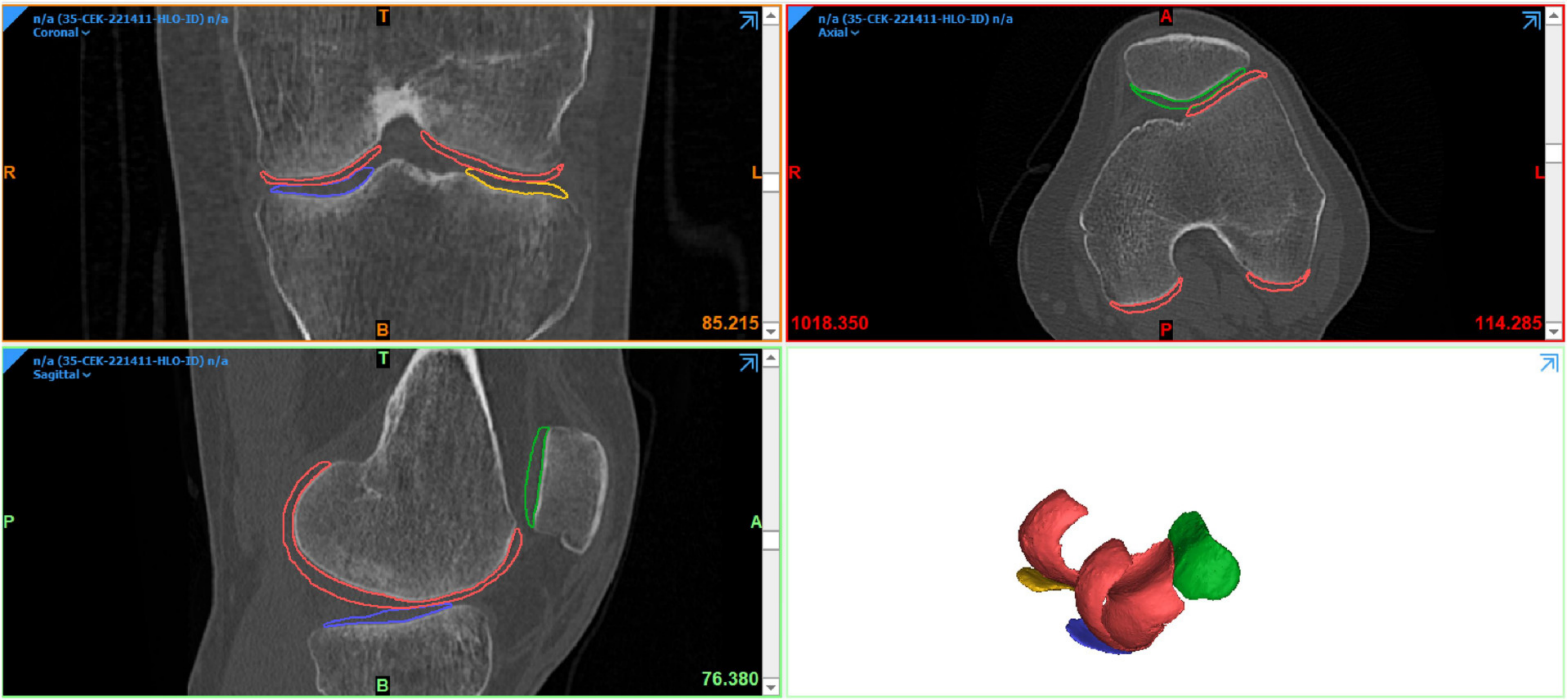
Coronal, axial, and sagittal view of segmented femoral (red), tibial (blue and yellow) and patellar (green) cartilages registered onto CT and corresponding 3D model.

Image processing included a protocol of cartilage segmentation and registration described and validated in (19–21). Briefly, knee cartilages (femoral, lateral tibial, medial tibial, and patellar) were segmented on MRI and transferred onto CT through a landmark-based image registration. The MRI segmentations were obtained using a semi-manual approach combining intensity-based thresholding with manual contour refinement, and subsequently aligned to CT via anatomical landmarks with minor adjustments to ensure spatial consistency. This enabled direct assessment of cartilage radiodensity on CT, expressed in Hounsfield units (HU). Three-dimensional (3D) parts were also created from the segmentations, to study morphological features. Figure 1 shows cartilage segmentations reported on the CT in the three main views (axial, coronal, and sagittal) and the relative 3D reconstructions. For each cartilage, the following features were extracted: average and standard deviation of HU values, as well as volume and surface data from the 3D models (Table 1). These features were selected because previous studies have demonstrated their ability to reliably distinguish between control and diseased groups (19, 22, 23). Building on this evidence, we aimed to further investigate their potential to refine disease category stratification. Materialise Mimics software was used to process the images and retain the features.

**Table 1:** List of features.

Feature category	ROI-specific feature
Avg Density (HU)	FemCart Avg Density
	LatTibCart Avg Density
	MedTibCart Avg Density
	PatCart Avg Density
StDev Density (HU)	FemCart StDev Density
	LatTibCart StDev Density
	MedTibCart StDev Density
	PatCart StDev Density
Volume/Surface (mm)	FemCart Vol/Surf
	LatTibCart Vol/Surf
	MedTibCart Vol/Surf
	PatCart Vol/Surf

### 2.3 Semi-supervised learning labeling strategy

#### 2.3.1 Semi-supervised learning and specialists’ evaluation

Semi-Supervised Learning (SSL) is a ML technique that lies between supervised (where labeled data is provided) and unsupervised learning (where no labeled data is available). It addresses the challenge of limited labeled data (often costly, time-consuming, or requiring expert and/or subjective judgment) by training models on a small labeled dataset alongside a larger set of unlabeled data. The assumption is that unlabeled data contains valuable structural patterns which, when combined with labeled examples, improve the model’s generalization and efficiency. SSL is frequently used to infer labels for unlabeled data by uncovering underlying relationships and patterns.

Since clinical records, pain reports, or symptom-based diagnoses are not suitable as objective ground truth for structural degeneration, we decided to use imaging-based evaluation to obtain a reliable initial labeled subset. Therefore, instead of labeling the entire dataset, we selected a limited number of scans to be reviewed by specialists. These expert-validated cases served as the seed labels for the SSL algorithm.

A team of specialists, composed of radiologists, an orthopedic surgeon, and engineers, reviewed 36 MRI and CT scans of the knee joints without any prior knowledge of the patient’s medical history or symptoms. The evaluation was conducted according to the following criteria:

- Score 0 (Healthy knee joint): no abnormalities on either CT or MRI
- Score 1 (Early degeneration): signs of osteophytes and/or subchondral sclerosis on CT; signs of chondral irregularity and/or edema on MRI
- Score 2 (Advanced degeneration): large osteophytes, severe subchondral sclerosis and cysts on CT; almost or complete disappearance of joint cartilage and fluid in joint on MRI

In the end, they agreed on the following:

- Class 0: 9 instances
- Class 1: 11 instances
- Class 2: 16 instances

The number of labeled samples (36, approximately 27% of the dataset) reflects a practical balance between expert effort and methodological requirements. Each labeled case required assessment of both MRI and CT scans by a team of specialists, making large-scale manual labeling prohibitively time-consuming. The goal was to provide a sufficiently diverse set of expert-validated seeds that captured clear structural exemplars of the three degeneration stages while keeping the expert review manageable. The 36 selected cases offered adequate representation of healthy, early, and advanced degeneration and were sufficient to initialize the graph-based SSL algorithms, which rely on the structural patterns present in the unlabeled data rather than on large volumes of annotated samples.

#### 2.3.2 Label propagation and label spreading

Label Propagation (LP) (24) and Label Spreading (LS) (25) are graph-based SSL techniques particularly useful for small datasets with limited labeled data, as in our case. They assume that the structure of the unlabeled data reflects the class structure and use a small set of labeled nodes (seeds) to propagate their labels to nearby unlabeled nodes via graph-encoded similarities. Both methods construct a similarity graph and use a propagation matrix to iteratively diffuse label information across the graph, with labeled nodes guiding the process. The two algorithms differ in how they modify the similarity matrix and apply clamping: LP uses hard clamping (keeping original labels fixed), whereas LS allows a degree of flexibility (therefore, it can change initial seeds), by minimizing a loss function with regularization, making it more robust to noise (26). The *sklearn.semi_supervised* scikit-learn Python library was used to implement the two algorithms.

#### 2.3.3 Labeling pipeline

Out of the 36 labeled samples, 9 (3 per class) are randomly excluded from the dataset to simulate unlabeled data, which will be used as a test set to validate the labeling protocol. The remaining 27 labeled instances are distributed as follows:

- Class 0: 6 instances
- Class 1: 8 instances
- Class 2: 13 instances

Both LP and LS were applied independently to the full set of 124 subjects, using the 27 labeled ones as seeds. The algorithms used the radial basis function (RBF) kernel (Equation 1), with γ=10. This value was chosen after testing γ from 0.5 to 20 and evaluating label distributions by average confidence and entropy. As γ increased, the label distributions became sharper: confidence increased while entropy decreased, demonstrating that the models became more certain as nearby labeled points had a stronger and more localized influence. Around γ=10, both models reached stable labeling, with minimal changes at higher values. Thus, γ=10 was selected as a balance, providing strong, stable, and confident labeling while retaining a neighborhood influence broad enough to capture patterns beyond very local structures.

$$\mathrm{RBF}=K(x,y)=\exp\left(-\gamma \|x-y{\|}^{2}\right)$$ (1)

*K* is the kernel function and it measures the similarity between points *x* and *y*. It returns a value between 0 and 1. When *x* and *y* are very close, $\|x-y{\|}^{2}$ is small, so *K* is close to 1 (high similarity). When *x* and *y* are far apart, $\|x-y{\|}^{2}$ is large, so *K* is close to 0 (low similarity). Higher γ means the kernel focuses more on points closer to each other (more localized influence), while lower γ means the influence spreads more broadly.

The label distributions produced by LS and LP models were combined by summing their respective class probabilities. The final predicted label (pseudo-label ${\hat{y}}_{i}$) for each sample i was then determined by selecting the class c with the highest combined probability (Equation 2).

$${\hat{y}}_{i}={\arg\max}_{c\in \{0,1,2\}}\left(P_{LS}(c|i)+P_{LP}(c|i)\right)$$ (2)

where $P_{LS}(c|i)$ and $P_{LP}(c|i)$ represent the probabilities of assigning the sample i to class c by the LS and LP models, respectively.

#### 2.3.4 Supervised learning and evaluation metrics

Supervised learning was applied to the new labeled dataset (healthy vs early degenerate vs late degenerate). Models were trained on a combined set of 124 samples (27 expert-labeled + 97 pseudo-labeled) and evaluated on the 9 held-out expert-labeled samples. To ensure robustness and mitigate variability from relying on a small set of expert labels (36 in total), the entire pipeline (from test split to label propagation and supervised training) is repeated 10 times with different random selections of 9 test subjects. This approach is a form of Monte Carlo cross-validation (27) (also known as repeated random subsampling), where multiple randomized train/test splits are used to better estimate generalization performance. To avoid data leakage, the 9 held out expert label saples are completley external from the training process, however to ensure generalizability these 9 samples differ at every iteration.

**Figure 2:**
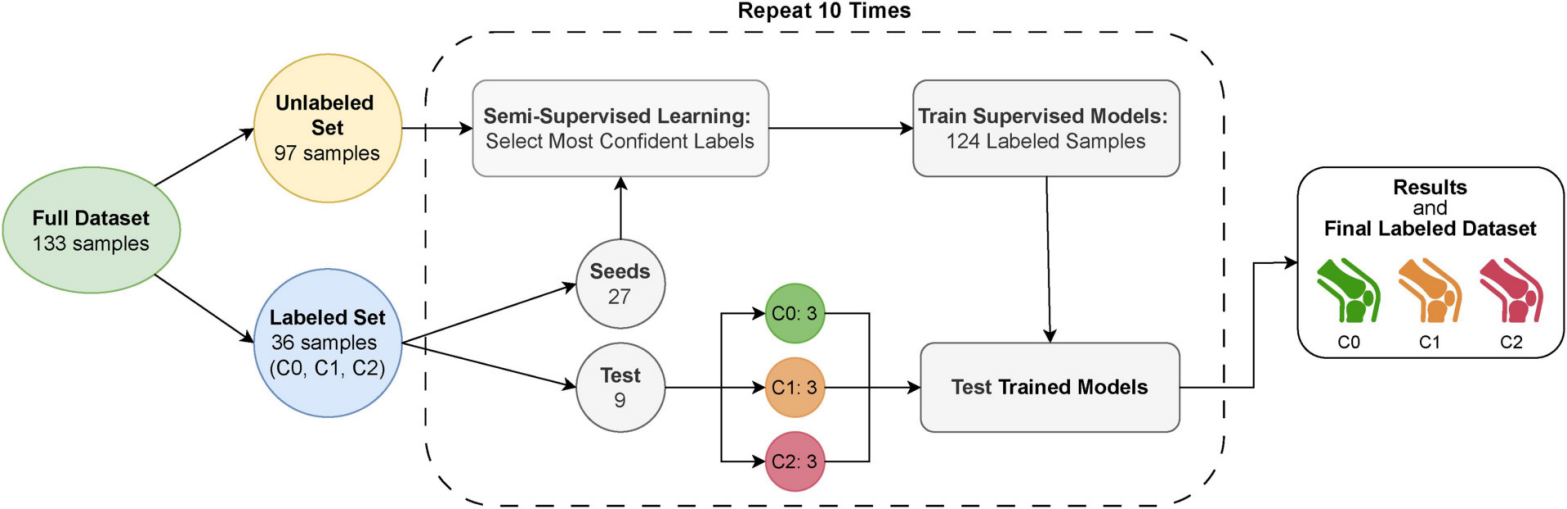
Workflow of the Labeling Strategy of Class 0 (C0, healthy), Class 1 (C1, early deg), Class 2 (C2, late deg).

By aggregating results across these 10 repetitions, the evaluation captures both central tendency and variability, yielding a more reliable assessment of the model’s predictive performance. Figure 2 gives an overview of the entire workflow for the labeling strategy described so far.

We assessed the following supervised learning algorithms: Support Vector Machine (SVM), Logistic Regression (LR), Random Forest (RF), Gradient Boosting (GB), K-Nearest Neighbors (KNN). All models were tuned using grid search with 5-fold cross-validation on the training data. For SVM, LR and KNN, features were scaled using StandardScaler to ensure consistent performance. The best-performing hyperparameters were selected based on average cross-validation scores. The hyperparameter grids used for tuning are shown in Table 2. Performance metrics included overall accuracy, weighted precision, recall, and F1 score, as well as per-class scores to evaluate class-wise performance.

**Table 2:** Hyperparameter grids used for cross-validated grid search.

Classifier	Hyperparameter Grid
SVM	C∈{0.1,1,10,100},kernel∈{linear,rbf}
LR	C∈{0.01,0.1,1,10,100}
RF	n\_estimators∈{10,50,100},max\_depth∈{3,5,10,None},min\_samples\_split∈{2,5,10}
GB	n\_estimators∈{10,50,100},learning\_rate∈{0.01,0.1,1},max\_depth∈{3,5,10}
KNN	k∈{3,5,7,9},weights∈{uniform,distance}

Metrics for AI in healthcare are more commonly defined in binary classification tasks (28). However, they can be easily extended to multi-class cases such as the one analyzed. Accuracy is defined as the ratio of correctly classified samples over the total instances. The other metrics are based on the evaluation of four values:

- true positive (TP), the number of samples classified as belonging to class X, correctly,
- true negative (TN), the number of samples classified as not belonging to class X, correctly,
- false positive (FP), the number of samples classified as belonging to class X, wrongly,
- false negative (FN), the number of samples classified as not belonging to class X, wrongly.

Precision is defined as the ratio of correctly predicted positive cases to all cases predicted as positive for that class (c), measuring the model’s accuracy in identifying true positives among its positive predictions (Equation 3).

$${\text{Precision}}_{c}=\frac{{\mathrm{TP}}_{c}}{{\mathrm{TP}}_{c}+{\mathrm{FP}}_{c}}$$ (3)

In case of an unbalanced dataset or multiclass classification tasks, weighted precision (Equation 4) is the mean of each class precision weighted by the number of instances ($N_{c}$) belonging to that class, among all the classes (C).

$$\text{Weighted Precision}=\frac{1}{N}\sum_{c=0}^{C}\left({\text{Precision}}_{c}\times N_{c}\right)$$ (4)

Recall (or sensitivity) is the ratio of correctly predicted positive cases to all actual positive cases, measuring the model’s ability to find all relevant instances in the data (Equation 5).

$${\text{Recall}}_{c}=\frac{{\mathrm{TP}}_{c}}{{\mathrm{TP}}_{c}+{\mathrm{FN}}_{c}}$$ (5)

Weighted recall is evaluated in a similar way to weighted precision (Equation 6).

$$\text{Weighted Recall}=\frac{1}{N}\sum_{c=0}^{C}\left({\text{Recall}}_{c}\times N_{c}\right)$$ (6)

Finally, the F1 score is defined as the harmonic mean of precision and recall, and it is commonly used to summarize both precision and recall in a single metric. It is usually more reliable than accuracy in cases of class imbalance. Therefore, we calculated the weighted F1 score as the harmonic mean of weighted recall and weighted precision.

### 2.4 Statistical analysis

#### 2.4.1 Label stability

To assess label stability across the ten independent runs, we computed some metrics:

1. Per-sample agreement: For each unlabeled sample (the set of 97 data points), it checks how often it got the same label across the 10 runs.
2. Fleiss’ kappa: widely utilized in the medical field, it is a statistical measure used to evaluate the reliability of agreement among multiple raters (more than two) that classify items into categorical outcomes. It quantifies the extent of agreement among several raters beyond what would be expected by chance (29).
3. Entropy: it measures the uncertainty of each sample across the runs; the lower the entropy (the lower the uncertainty), the more stable the label.

#### 2.4.2 Group comparisons

As a first evaluation of the newly created SSL labels, inferential statistical tests were employed to evaluate the differences among the three categories: healthy, early degeneration, and late degeneration. Although the dataset was labeled using ML methods, traditional inferential statistics were essential for validating group-level differences. Shapiro-Wilk test was conducted to assess normality, and one-way ANOVA (30) and the Kruskal-Wallis tests (31) were used to assess group differences in normally and non-normally distributed variables, respectively. The *p*-values obtained from these tests provided a quantitative measure of statistical significance on the extracted features. To further explore specific intergroup differences, post hoc analyses using Tukey’s HSD (32) and Dunn’s (33) test were conducted, controlling for multiple comparisons. This hybrid approach of ML and statistical testing strengthens the reliability of the findings and supports the clinical relevance of the categorized outcomes.

## 3 Results

### 3.1 Three-class classification performances

In each independent run, 124 samples were used for training the classification algorithms, and performances were computed on the test set composed of the 9 held-out samples. The results were combined from the ten runs. Table 3 reports the mean and standard deviation of the ten runs for each metric and model on the three-class problem. Tables 4–6 show the corresponding results for classes 0 (healthy), 1 (early degeneration), and 2 (severe degeneration).

**Table 3:** Comparison of 3 classes algorithm’s metrics with mean ± standard deviation on ten runs, sorted by F1 Weighted score.

Model	Accuracy	Precision weighted	Recall weighted	F1 weighted
SVM	0.84±0.11	0.88±0.08	0.84±0.11	0.84±0.11
LR	0.82±0.12	0.87±0.10	0.82±0.12	0.81±0.13
KNN	0.80±0.10	0.83±0.11	0.80±0.10	0.79±0.11
RF	0.79±0.11	0.80±0.15	0.79±0.11	0.77±0.14
GB	0.76±0.17	0.76±0.20	0.76±0.17	0.74±0.18

**Table 4:** Performance metrics for Class 0 across models, reported as mean ± standard deviation.

Class 0			
Model	Precision	Recall	F1-score
SVM	0.95±0.11	0.97±0.11	0.95±0.08
LR	0.88±0.15	0.87±0.17	0.86±0.13
KNN	0.95±0.11	1.00±0.00	0.97±0.06
RF	0.88±0.16	0.93±0.14	0.89±0.12
GB	0.85±0.21	0.93±0.14	0.89±0.18

**Table 5:** Performance metrics for Class 1 across models, reported as mean ± standard deviation.

Class 1			
Model	Precision	Recall	F1-score
SVM	0.89±0.17	0.73±0.21	0.78±0.15
LR	0.78±0.17	0.93±0.14	0.83±0.12
KNN	0.82±0.20	0.70±0.25	0.71±0.16
RF	0.76±0.14	0.80±0.23	0.75±0.12
GB	0.75±0.18	0.73±0.21	0.73±0.16

**Table 6:** Performance metrics for Class 2 across models, reported as mean ± standard deviation.

Class 2			
Model	Precision	Recall	F1-score
SVM	0.81±0.18	0.83±0.24	0.79±0.16
LR	0.94±0.12	0.67±0.27	0.74±0.19
KNN	0.73±0.20	0.70±0.29	0.68±0.20
RF	0.77±0.32	0.63±0.29	0.67±0.26
GB	0.68±0.31	0.60±0.34	0.61±0.30

The highest overall performance was obtained by SVM, with an F1-Weighted of 0.84 ± 0.11, followed by LR at 0.81 ± 0.13. The lowest-performing model was GB, with 0.74 ± 0.18. Accuracy values followed a similar trend, ranging from 0.76 (GB) to 0.84 (SVM). Weighted precision reached its highest values with SVM (0.88 ± 0.08) and LR (0.87 ± 0.10), while recall-weighted was also led by SVM (0.84 ± 0.11) and LR (0.82 ± 0.12).

At the class level, class 0 consistently reached the highest scores across models, with KNN achieving an F1 of 0.97 and SVM 0.95. Class 1 results were lower overall but remained stable, with LR performing best (F1 = 0.83) followed by SVM (0.78). For class 2, SVM obtained the highest F1 (0.79), while LR showed a strong precision value (0.94) accompanied by lower recall (0.67).

Taken together, these results show that all algorithms reached acceptable performance, with class 0 producing the most consistent metrics and higher variability observed in classes 1 and 2.

### 3.2 Label stability

Agreement per-sample across the ten runs was calculated; the average and standard deviation among the runs are 0.911 ± 0.136, indicating that, on average, 91% of the runs agree on each sample’s label, with a slight variability. Fleiss’ Kappa is 0.781, indicating strong agreement between runs beyond chance. Finally, the mean entropy across runs is 0.283 with a standard deviation of 0.385; this indicates that most samples have low uncertainty among the different runs. These outcomes demonstrate that the SSL pipeline provided reliable and consistent labels across runs, with high agreement and low entropy indicating stability across different seed initializations.

### 3.3 Re-labeled dataset: statistical analysis and distributions

**Figure 3:**
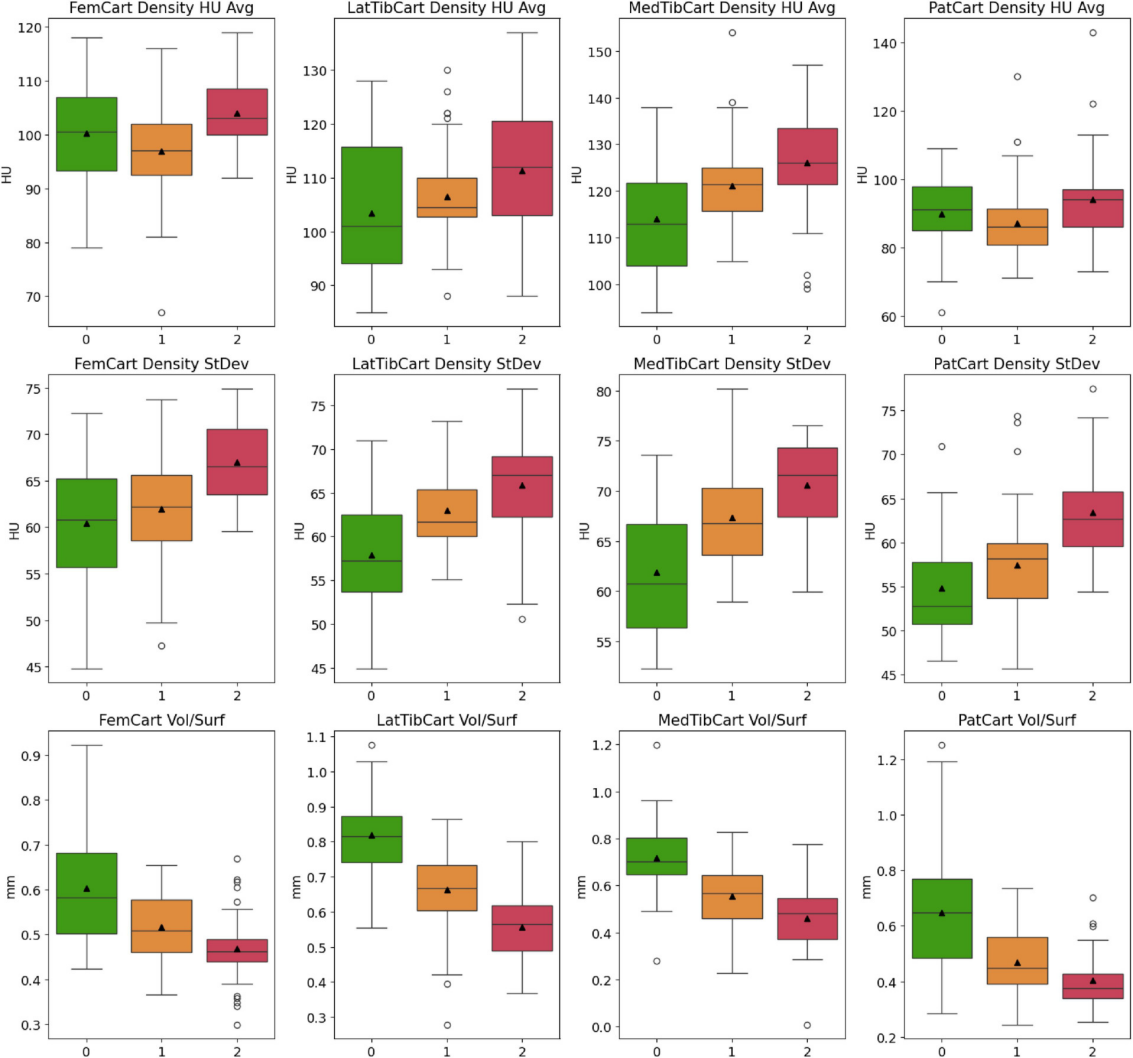
Boxplot distributions of the full labeled dataset for each feature, divided by classes 0, 1, and 2 (along *x*-axis); *y*-axis represents HU for density features and mm for Vol/Surf ones.

The unlabeled samples were finally labeled through majority agreement between the ten runs. The final full dataset, including the originally labeled 36 samples and the new machine-labeled 97 ones, was used to evaluate statistically significant differences on the considered features among the three classes. ANOVA/Kruskal-Wallis tests (parametric/non-parametric) showed significant *p*-values for each evaluated feature; therefore, a post hoc analysis was conducted. For Tukey’s test, p-values adjusted with Bonferroni correction were considered. Results are reported in Table 7 and show that the considered features discriminated between healthy, early degeneration, and late degeneration, with the strongest separation observed for the density standard deviation of both tibial cartilages and the volume-over-surface features. Figure 3 illustrates these differences, displaying the distributions of all features across the three classes and highlighting clear progressive trends. For volume-to-surface ratio features, a decreasing trajectory is observed from healthy to severe degeneration. Tibial cartilages in particular displayed highly significant reductions (p<0.001).

**Table 7:** Post Hoc analysis results for cartilage metrics (*P*-values rounded to 3 significant digits).

Feature	Test	Groups	*P*-value	Reject
FemCart Density HU Avg	Dunn	0 vs. 1	0.191	False
		0 vs. 2	0.105	False
		1 vs. 2	0.000	True
LatTibCart Density HU Avg	Dunn	0 vs. 1	0.416	False
		0 vs. 2	0.008	True
		1 vs. 2	0.215	False
MedTibCart Density HU Avg	Dunn	0 vs. 1	0.022	True
		0 vs. 2	0.000	True
		1 vs. 2	0.055	False
PatCart Density HU Avg	Dunn	0 vs. 1	0.067	False
		0 vs. 2	1.000	False
		1 vs. 2	0.007	True
FemCart Density StDev	Tukey HSD	0 vs. 1	0.343	False
		0 vs. 2	0.000	True
		1 vs. 2	0.000	True
LatTibCart Density StDev	Tukey HSD	0 vs. 1	0.000	True
		0 vs. 2	0.000	True
		1 vs. 2	0.043	True
MedTibCart Density StDev	Tukey HSD	0 vs. 1	0.000	True
		0 vs. 2	0.000	True
		1 vs. 2	0.011	True
PatCart Density StDev	Dunn	0 vs. 1	0.123	False
		0 vs. 2	0.000	True
		1 vs. 2	0.000	True
FemCart Vol/Surf	Tukey HSD	0 vs. 1	0.000	True
		0 vs. 2	0.000	True
		1 vs. 2	0.060	False
LatTibCart Vol/Surf	Dunn	0 vs. 1	0.000	True
		0 vs. 2	0.000	True
		1 vs. 2	0.001	True
MedTibCart Vol/Surf	Dunn	0 vs. 1	0.000	True
		0 vs. 2	0.000	True
		1 vs. 2	0.012	True
PatCart Vol/Surf	Dunn	0 vs. 1	0.000	True
		0 vs. 2	0.000	True
		1 vs. 2	0.031	True

Density-related features exhibited more complex trends. Average densities varied inconsistently across different groups. Some sites, such as the femoral and patellar areas, showed early decreases followed by later increases, while tibial cartilage demonstrated a consistent upward trend. Significant differences were mainly observed between classes 1 and 2 in the femoral and patellar sites, and between healthy and degenerated knees in the tibial regions. In contrast, the standard deviation of density provided clearer distinctions, revealing significantly higher and increasing heterogeneity in the diseased groups, with significant differences detected in nearly all pairwise comparisons.

Overall, both geometric (volume-to-surface) and heterogeneity (density standard deviation) features showed the most robust ability to separate the three groups, while mean density values displayed greater variability across sites.

## 4 Discussion

The image-extracted features and the defined SSL pipeline used in this work proved their ability in labeling the dataset in three different classes according to the severity and progression of the disease, by capturing consistent patterns within the data.

### 4.1 SSL pipeline

#### 4.1.1 Seed set size, composition, and label stability

The developed framework relied on 36 expert-labeled samples and yielded considerable results. Although 27% of the dataset may appear small, this number represents an effective balance between the effort required for expert annotation and the need for a sufficiently informative seed set. Using a substantially larger number of labeled cases would shift the approach toward a traditional supervised setting, reducing the added value of SSL and reintroducing the practical burden of extensive manual annotation. Conversely, a markedly smaller seed set would not sufficiently represent the heterogeneity of the three degeneration stages, leading to unstable propagation and unreliable pseudo-labels, and would also make meaningful validation impossible due to an insufficient test set. The chosen number of 36 labeled cases therefore ensured an appropriate compromise: large enough to provide clinically reliable anchors for SSL, yet small enough to preserve the advantages of semi-supervision in scenarios where expert annotation is limited.

In addition to the number of labeled seeds, the composition of the seed set also deserves consideration, particularly the slight imbalance across the three expert-defined classes (9 healthy, 11 early degeneration, 16 advanced degeneration). Although not perfectly uniform, this distribution had limited influence on the SSL process. Graph-based methods such as Label Propagation and Label Spreading rely primarily on geometric similarity within the feature space rather than class frequencies. As long as each class includes a sufficient number of clear, structurally consistent exemplars, the propagation remains stable. This is supported by the high per-sample agreement (0.911 ± 0.136) across the ten runs, indicating that most samples receive consistent labels regardless of seed initialization or test split. Similarly, Fleiss’ Kappa of 0.781 confirms substantial agreement beyond chance, while the low mean entropy (0.283 ± 0.385) suggests that the model is generally confident in its assignments. Therefore, the mild imbalance did not introduce bias or instability. Instead, the separability of the underlying imaging features played a more decisive role than the exact numerical proportion of seeds.

Together, these metrics indicate that the labeling procedure is reliable, although higher entropy in a subset of samples highlights regions where the strategy may struggle with borderline or ambiguous cases. Given the intrinsic subjectivity that characterizes degenerative disease assessment, a degree of disagreement or uncertainty is expected, even among domain experts.

#### 4.1.2 Classification performances

The three-class classification results highlight clear differences in performance across models and classes. At the global level, SVM consistently outperforms the other approaches, achieving the highest weighted F1-score (0.84 ± 0.11), followed by LR (0.81 ± 0.13). Even the lowest-performing model, GB, maintains a reasonable F1-score (0.74 ± 0.18), suggesting that all tested algorithms are able to capture relevant information from the data despite the small sample size and inherent variability across runs.

When analyzing class-specific performances, clear differences emerge. Class 0 (healthy) is identified with high reliability across all models, with near-perfect recall and precision in some cases (e.g., KNN with F1 = 0.97, SVM with F1 = 0.95). This suggests that healthy samples occupy a more distinct region in feature space, making them easier to separate. In contrast, Class 1 (early degeneration) proves more challenging. LR achieves the best balance here (F1 = 0.83), with particularly strong recall, suggesting it is better able to capture subtle patterns indicative of early-stage disease. Class 2 (severe degeneration) also shows variability across models: SVM maintains balanced performance (F1 = 0.79), whereas LR reaches high precision (0.94) but sacrifices recall (0.67), indicating a more conservative labeling tendency that avoids false positives but risks under-detection of severe cases.

These findings show how different models emphasize distinct trade-offs between precision and recall, particularly for the more ambiguous diseased classes. The broader variability across runs, especially in Classes 1 and 2, reflects the difficulty of discriminating progressive disease stages and the challenges of small-sample biomedical evaluation. Nonetheless, the repeated experiments demonstrate that certain models, such as SVM, remain robust across different splits, while others, such as LR, show complementary strengths that could be valuable in ensemble approaches. Overall, healthy samples are reliably identified, while distinguishing between early and severe degeneration is more challenging, although still feasible. AI in pain assessment must be interpretable, multimodal, and clinically viable (34), principles our SSL pipeline addresses with multimodal imaging and reproducibility. Prior work also notes that while healthy controls are easily separated, early- and late-stage KOA are harder to distinguish due to overlapping features and stages’ gradual transitions (35). These findings represent a crucial step in advancing severity stratification beyond a binary classification by utilizing multimodal imaging features to effectively capture the complexities of progressive disease.

#### 4.1.3 Comparison with existing literature

Most studies on knee osteoarthritis classification rely on fully supervised methodologies applied to X-ray or MRI data, typically requiring large, fully annotated datasets. These approaches commonly focus on binary classification or grading based on established radiographic scales and use cartilage-derived morphological or radiomic features combined with conventional ML models (36–38). More advanced radiomics-based frameworks have demonstrated high diagnostic and prognostic performance by incorporating cartilage and subchondral bone features (39, 40). However, they remain inherently dependent on extensive labeled data and predefined grading systems. Additionally, they mirror clinical scoring conventions instead of providing objective measures of cartilage structure, which can lead to inter-observer variability.

A direct comparison with a purely supervised approach within our dataset is not feasible, as only 36 expert-labeled samples are available, which is insufficient to train a reliable three-class supervised model and would lead to unstable and non-generalizable results. This limitation reflects a realistic clinical scenario, where obtaining large, consistently annotated datasets is challenging due to the cost, time, and subjectivity associated with expert labeling.

Within this context, the proposed semi-supervised framework provides a practical alternative by leveraging both labeled and unlabeled data to learn meaningful structure in the feature space. Unlike prior semi-supervised methods, which are often limited to tasks such as cartilage grading or segmentation (12), the present approach directly addresses multi-class structural stratification using multimodal MRI and CT-derived features. This allows the model to capture complementary information on cartilage morphology and density while reducing dependence on subjective radiographic grading systems.

Overall, rather than competing directly with fully supervised models trained on large annotated cohorts, the proposed method addresses a different and clinically relevant setting: limited-label scenarios. In this sense, it complements existing literature by demonstrating that reliable and interpretable classification of knee degeneration can be achieved even when only a small expert-labeled subset is available.

### 4.2 Statistical results and clinical interpretation

The final dataset of 133 samples validated the employed labeling strategy. As shown in Table 7, many features reject the null hypothesis, indicating that the SSL pipeline captures both structural (density) and geometrical (volume/surface) changes across healthy, early, and severe degeneration. This supports that the data-driven stratification reflects biological and morphological differences rather than being arbitrary. Figure 3 further shows that labeled samples follow ascending or descending trends for nearly every feature, consistent with expected degeneration progression. This strengthens ML interpretability, suggesting models separate classes statistically while aligning with known pathophysiological mechanisms.

Most Vol/Surf features effectively differentiate healthy, early, and late degeneration, with the femoral cartilage showing some difficulty distinguishing the two degeneration stages, though remaining significant when comparing healthy and degenerative cases. The volume-to-surface ratio indicates that higher values reflect greater tissue volume relative to surface area. Healthy cartilage is thick, smooth, and evenly distributed, yielding a high Vol/Surf ratio. Osteoarthritic cartilage, conversely, is thinner, fibrillated, and irregular, reducing volume while increasing effective surface area, and thus lowering the Vol/Surf ratio. Multiple studies (41, 42) confirm that cartilage volume is significantly reduced in KOA across knee compartments and that tibial surface area increases, particularly in the presence of osteophytes (bony outgrowths). Early KOA is associated with concurrent cartilage volume loss and bone remodeling (42, 43), and longitudinal studies confirm progressive volume loss (44, 45). Overall, the downward Vol/Surf trends support KOA pathophysiology and suggest this ratio as a sensitive marker for degeneration.

Density results reveal an interesting trend. Average densities of femoral and patellar regions are less predictive, particularly among healthy and advanced cases, though significant differences exist between classes 1 and 2. This suggests density initially decreases, then increases. While this may appear counterintuitive, several mechanisms may explain it. Early KOA degrades the softer, water-rich regions of cartilage, which are mechanically weaker and less dense (46). As these regions are lost, remaining cartilage appears denser on imaging due to exclusion of degraded areas, not because healthy tissue densifies. Late-stage KOA also shows increased mineralization and crystal accumulation in the calcified cartilage layer (47, 48), raising HU values without improving mechanical function (46, 48). Healthy cartilage retains a hydrated, proteoglycan-rich matrix, explaining lower density and variability. Technical factors like joint space narrowing may also affect segmentation. Site-specific differences clarify trends: tibial cartilage shows a more pronounced density increase, possibly due to meniscal protection of femoral cartilage and higher mechanical load on the tibia. Early decreases in femoral and patellar cartilage density may reflect lower mechanical stress, with tibial and trochlear moduli declining earlier (49). Standard deviation (density heterogeneity) provides more consistent separation across groups. Almost all sites, including tibial and femoral cartilage, show significant differences in StDev between healthy, early, and late KOA, indicating greater intra-cartilage heterogeneity in KOA. This aligns with MRI and histopathological studies reporting higher variability in water content, matrix organization, and density in degenerating cartilage (50). It is also important to consider that degeneration is not occurring all at once throughout the entire knee compartment, but rather progresses gradually and may affect different regions to varying degrees over time. Notably, heterogeneity may be a more sensitive marker than mean density, particularly for early detection.

Overall, these results highlight that while mean density trends are site-dependent and sometimes sophisticated to understand, variability measures consistently reflect degeneration. Combined with Vol/Surf features, which robustly separate all classes, this supports the multifactorial nature of KOA and validates the clinical relevance of the new three-class labels derived from multimodal imaging.

It is important to note that only a small portion of the potentially extractable data was utilized for this study. This approach was taken to enhance usability, as medical imaging software such as Mimics enables the easy extraction of smaller datasets. Additionally, it helps to address the limitations of our computational resources, as the analyses performed require significant processing power. However, radiomic features have been extracted from similar imaging datasets by Angelone et al. (39), specifically first-order, 2D/3D shape, and texture features, including wavelet-derived variants. With an accuracy of 90%, texture (especially wavelet-GLSZM) and shape features emerged as the strongest predictors of cartilage degeneration, in line with the trends observed in the present study. Integrating these radiomic descriptors, either the full set or a subset of the most informative ones, into our current framework could further strengthen its predictive capacity, enabling not only the detection of disease presence but also a more nuanced stratification of degeneration severity.

The proposed multimodal SSL-driven framework has the potential to support clinical practice by providing objective, reproducible, and image-based stratification of knee degeneration independent of pain or symptom variability. These quantitative biomarkers may assist radiologists and orthopedic surgeons in detecting early structural changes, facilitating earlier intervention and improving patient outcomes. Moreover, the reproducibility of the SSL-derived labels suggests that the method could be integrated into longitudinal studies or clinical trials as a standardized metric, reducing the subjectivity inherent in currently adopted grading systems such as Kellgren–Lawrence or Ahlbäck.

### 4.3 Limitations

This study has some limitations that should be acknowledged. First, the dataset was relatively small (133 knees, with only 36 expert-labeled), which may limit the generalizability of the results and increase the variability in model performance. In particular, the cohort was acquired at a single center using standardized acquisition protocols; this may restrict the applicability of the proposed models to other populations, imaging protocols, and clinical settings.

Additionally, class imbalance among the degeneration stages may have affected the stability and accuracy of the model, particularly for early and severe cases.

The reliance on pseudo-labels also introduces potential bias and uncertainty, especially for borderline samples. Furthermore, the lack of longitudinal data prevented an assessment of disease progression.

It is important to consider that, although categorizing KOA into healthy, early, and late degeneration provides an interpretable progression model, it oversimplifies the continuous nature of KOA. Osteoarthritis does not progress in discrete stages, and patients often fall between stages. Some misclassifications observed in ML performance and label variability likely reflect these biological overlaps.

Finally, although the framework showed strong performance, it is based on handcrafted features and classical machine learning models, which may not fully capture complex spatial patterns in imaging data. More advanced deep learning approaches could potentially improve performance through end-to-end feature learning, but they typically require substantially larger annotated datasets and higher computational resources. Future work should therefore explore these approaches alongside validation in larger and more diverse cohorts and real-world clinical settings to ensure robustness and applicability.

## 5 Conclusions

This study introduces a semi-supervised learning framework for characterizing different stages of knee osteoarthritis using objective cartilage features derived from combined MRI and CT scans, rather than relying on subjective symptoms such as pain. By integrating expert-validated labels with graph-based propagation methods, the approach successfully uncovered latent structure within multimodal imaging data and produced stable class assignments representing healthy, early, and advanced degeneration. Despite the limited number of expert annotations, the SSL strategy generated consistent pseudo-labels with strong agreement across multiple runs, demonstrating its suitability for scenarios where ground truth is uncertain or costly to obtain. The resulting three-class dataset enabled robust supervised classification, with SVM and LR achieving the highest performance, confirming that meaningful structural differences can be captured even from relatively simple feature sets. Importantly, the statistical analyses showed that the SSL-derived classes correspond to biologically interpretable trends in cartilage morphology and density variation. Future work should focus on developing more granular, continuous severity metrics that better reflect the progressive nature of KOA. Longitudinal validation in larger and more diverse cohorts will be essential to establish these imaging-derived features as reliable biomarkers for disease monitoring, prognosis, and treatment stratification. Additionally, incorporating clinical outcomes and expanding the framework to include automatic feature extraction from whole-image data could further enhance its clinical relevance and translational potential.

## Data Availability

The raw data supporting the conclusions of this article will be made available by the authors, without undue reservation.

## References

[1] Solomon CG Sharma L. Osteoarthritis of the knee. N Engl J Med. (2021) 384(1):51–9. 10.1056/nejmcp1903768 33406330

[2] Gelber AC. Knee osteoarthritis. Ann Intern Med. (2024) 177:ITC129–44. 10.7326/ANNALS-24-01249 39250809

[3] Katz JN Arant KR Loeser RF. Diagnosis and treatment of hip and knee osteoarthritis: a review. JAMA. (2021) 325(6):568–78. 10.1001/jama.2020.22171 33560326 PMC8225295

[4] Mahmoudian A Lohmander LS Mobasheri A Englund M Luyten FP. Early-stage symptomatic osteoarthritis of the knee—time for action. Nat Rev Rheumatol. (2021) 17:621–32. 10.1038/s41584-021-00673-4 34465902

[5] Piccolo C Mallio C Vaccarino F Grasso R Zobel B. Imaging of knee osteoarthritis: a review of multimodal diagnostic approach. Quant Imaging Med Surg. (2023) 13:7582–95. 10.21037/qims-22-1392 37969633 PMC10644136

[6] Petersson I Boegård T Saxne T Silman A Svensson B. Radiographic osteoarthritis of the knee classified by the Ahlbäck and Kellgren & Lawrence systems for the tibiofemoral joint in people aged 35–54 years with chronic knee pain. Ann Rheum Dis. (1997) 56:493–6. 10.1136/ard.56.8.493 9306873 PMC1752423

[7] Wing N van Zyl N Wing M Corrigan R Loch A Wall C. Reliability of three radiographic classification systems for knee osteoarthritis among observers of different experience levels. Skeletal Radiol. (2021) 50:399–405. 10.1007/s00256-020-03551-4 32780155

[8] Johnston JD Masri BA Wilson DR. Computed tomography topographic mapping of subchondral density (CT-tomasd) in osteoarthritic and normal knees: methodological development and preliminary findings. Osteoarthritis Cartilage. (2009) 17:1319–26. 10.1016/j.joca.2009.04.013 19427927

[9] Cush JJ. Rheumatoid arthritis: early diagnosis and treatment. Med Clin North Am. (2021) 105(2):355–65. 10.1016/j.mcna.2020.10.006 33589108

[10] Rida MA Chandran V. Challenges in the clinical diagnosis of psoriatic arthritis. Clin Immunol. (2020) 214:108390. 10.1016/j.clim.2020.108390 32200113

[11] Huo J Si L Ouyang X Xuan K Yao W Xue Z, et al. A self-ensembling framework for semi-supervised knee cartilage defects assessment with dual-consistency. In: *Predictive Intelligence in Medicine*. Cham: Springer (2020). p. 200–9.

[12] Huo J Ouyang X Si L Xuan K Wang S Yao W, et al. Automatic grading assessments for knee MRI cartilage defects via self-ensembling semi-supervised learning with dual-consistency. Med Image Anal. (2022) 80:102508. 10.1016/j.media.2022.102508 35759870

[13] Belton N Lawlor A Curran KM. An ai system for continuous knee osteoarthritis severity grading: an anomaly detection inspired approach with few labels. Artif Intell Med. (2025) 167:103138. 10.1016/j.artmed.2025.103138 40449142

[14] Raisuddin AM Nguyen HH Tiulpin A. Deep semi-supervised active learning for knee osteoarthritis severity grading. In: *2022 IEEE 19th International Symposium on Biomedical Imaging (ISBI)*. (2022). p. 1–5. 10.1109/ISBI52829.2022.9761668

[15] Alshamrani HA Rashid M Alshamrani SS Alshehri AHD. Osteo-net: an automated system for predicting knee osteoarthritis from x-ray images using transfer-learning-based neural networks approach. Healthcare. (2023) 11. 1206 10.3390/healthcare11091206 37174748 PMC10178688

[16] Islam MS Rony M. CDK: a novel high-performance transfer feature technique for early detection of osteoarthritis. J Pathol Inform. (2024) 15:100382. 10.1016/j.jpi.2024.100382 38840834 PMC11152656

[17] Sohail M Azad MM Kim HS. Knee osteoarthritis severity detection using deep inception transfer learning. Comput Biol Med. (2025) 186:109641. 10.1016/j.compbiomed.2024.109641 39742824

[18] Morales EF Escalante HJ. Chapter 6—a brief introduction to supervised, unsupervised, and reinforcement learning. In: Torres-García AA, Reyes-García CA, Villaseíor-Pineda L, Mendoza-Montoya O, editors, *Biosignal Processing and Classification Using Computational Learning and Intelligence*. New York City: Academic Press (2022). p. 111–29. 10.1016/B978-0-12-820125-1.00017-8

[19] Ciliberti FK Guerrini L Gunnarsson AE Recenti M Jacob D Cangiano V, et al. CT- and MRI-based 3D reconstruction of knee joint to assess cartilage and bone. Diagnostics. (2022) 12(2):279. 10.3390/diagnostics12020279 35204370 PMC8870751

[20] Aubonnet R Ramos J Recenti M Jacob D Ciliberti F Guerrini L, et al. Toward new assessment of knee cartilage degeneration. Cartilage. (2023) 14:351–74. 10.1177/19476035221144746 36541701 PMC10601563

[21] Fox S Ciliberti FK Jönsson H Gargiulo P Recenti M. Impact of automated and manual segmentation errors on knee osteoarthritis classification using MRI-registered data on CT scans. Comput Med Imaging Graph. (2026) 131:102767. 10.1016/j.compmedimag.2026.102767 41985396

[22] Ciliberti FK Maruotto I Jónsson H Gíslason MK Gargiulo P. Multimetric evaluation of knee cartilage degeneration. In: *2024 IEEE International Conference on Metrology for eXtended Reality, Artificial Intelligence and Neural Engineering (MetroXRAINE)*. (2024). p. 1022–7. 10.1109/MetroXRAINE62247.2024.10795901

[23] Maruotto I Ciliberti FK Gargiulo P Recenti M. Feature selection in healthcare datasets: towards a generalizable solution. Comput Biol Med. (2025) 196:110812. 10.1016/j.compbiomed.2025.110812 40738051

[24] ZhuĞ X Ghahramanin Z. Learning from labeled and unlabeled data with label propagation. *ProQuest number: information to all users*. (2002).

[25] Bengio Y Le Roux N Delalleau O, Efficient Non-Parametric Function Induction in Semi-Supervised Learning. Montreal: CIRANO (2004).

[26] Song Z Yang X Xu Z King I. Graph-based semi-supervised learning: a comprehensive review. IEEE Trans Neural Networks Learn Syst. (2023) 34:8174–94. 10.1109/TNNLS.2022.3155478 35302941

[27] Xu QS Liang YZ. Monte carlo cross validation. Chemometr Intell Lab Syst. (2001) 56:1–11. 10.1016/S0169-7439(00)00122-2

[28] Hicks SA Strümke I Thambawita V Hammou M Riegler MA Halvorsen P. On evaluation metrics for medical applications of artificial intelligence. Sci Rep. (2022) 12:5979. 10.1038/s41598-022-09954-8 35395867 PMC8993826

[29] Zapf A Castell S Morawietz L Karch A. Measuring inter-rater reliability for nominal data—which coefficients and confidence intervals are appropriate? BMC Med Res Methodol. (2016) 16:93. 10.1186/s12874-016-0200-9 27495131 PMC4974794

[30] Chatzi A Doody O. The one-way ANOVA test explained. Nurse Res. (2023) 31:8–14. 10.7748/nr.2023.e1885 37317616

[31] McKight PE Najab J. Kruskal-wallis test. Corsini Encyclopedia Psychol. (2010) 1:1. 10.1002/9780470479216.corpsy0491

[32] Nanda A Mohapatra BB Mahapatra AP Mahapatra APK Mahapatra A. Multiple comparison test by Tukey’s honestly significant difference (HSD): do the confident level control type I error. Int J Stat Appl Math. (2021) 6:59–65. 10.22271/MATHS.2021.V6.I1A.636

[33] Dinno A. Nonparametric pairwise multiple comparisons in independent groups using Dunn’s test. Stata J. (2015) 15:292–300. 10.1177/1536867X1501500117

[34] Cascella M Ponsiglione AM Santoriello V Romano M Cerrone V Esposito D, et al. Expert consensus on feasibility and application of automatic pain assessment in routine clinical use. J Anesthesia Analg Crit Care. (2025) 5:29. 10.1186/s44158-025-00249-8 PMC12131339 40457422

[35] Mahmoudian A Lohmander L Jafari H Luyten F. Towards classification criteria for early-stage knee osteoarthritis: a population-based study to enrich for progressors. Semin Arthritis Rheum. (2021) 51(1):285–91. 10.1016/j.semarthrit.2020.11.002 33433364

[36] Cengizler Ç Kabakcı AG. A K-nearest neighbors-based classification approach for automated detection of knee osteoarthritis. Cukurova Med J. (2023) 48(2):715–22. 10.17826/cumj.1281955

[37] Cui T Liu R Jing Y Fu J Chen J. Development of machine learning models aiming at knee osteoarthritis diagnosing: an MRI radiomics analysis. J Orthop Surg Res. (2023) 18:375. 10.1186/s13018-023-03837-y 37210510 PMC10199595

[38] Fatema K Rony MAH Azam S Mukta MSH Karim A Hasan MZ, et al. Development of an automated optimal distance feature-based decision system for diagnosing knee osteoarthritis using segmented x-ray images. Heliyon. (2023) 9:e21703. 10.1016/j.heliyon.2023.e21703 38027947 PMC10665756

[39] Angelone F Ciliberti FK Tobia GP Jónsson Jr H Ponsiglione AM Gislason MK, et al. Innovative diagnostic approaches for predicting knee cartilage degeneration in osteoarthritis patients: a radiomics-based study. Inf Syst Front. (2025) 27:51–73. 10.1007/s10796-024-10527-5

[40] Fu J Mu L Dong D Li M Miao Z Huai X, et al. An MRI-based radiomics framework for early identification and progression stratification in knee osteoarthritis: data from the osteoarthritis initiative. BMC Musculoskelet Disord. (2025) 26:1018. 10.1186/s12891-025-09234-2 41174627 PMC12577384

[41] Ünalmış D Acer N Yılmaz S Tokpınar A Doğan S Demir H. The calculation of the femoral condyle cartilage volume and surface area in patients with osteoarthritis. J Clin Pract Res. (2020) 42:178–84. 10.14744/etd.2020.75768

[42] Ding C Garnero P Cicuttini F Scott F Cooley H Jones G. Knee cartilage defects: association with early radiographic osteoarthritis, decreased cartilage volume, increased joint surface area and type II collagen breakdown. Osteoarthritis Cartilage. (2005) 13(3):198–205. 10.1016/J.JOCA.2004.11.007 15727885

[43] Bonakdari H Pelletier J Abram F Martel-Pelletier J. A machine learning model to predict knee osteoarthritis cartilage volume changes over time using baseline bone curvature. Biomedicines. (2022) 10:1247. 10.3390/biomedicines10061247 35740270 PMC9220338

[44] Zhai G Pelletier J Liu M Aitken D Randell E Rahman P, et al. Activation of the phosphatidylcholine to lysophosphatidylcholine pathway is associated with osteoarthritis knee cartilage volume loss over time. Sci Rep. (2019) 9:9648. 10.1038/s41598-019-46185-w 31273319 PMC6609700

[45] Brisson N Wiebenga E Stratford P Beattie K Totterman S Tamez-Peña JG, et al. Baseline knee adduction moment interacts with body mass index to predict loss of medial tibial cartilage volume over 2.5 years in knee osteoarthritis. J Orthop Res. (2017) 35:2476. 10.1002/jor.23564 28323351

[46] Cooke M Lawless B Jones SW Grover L. Matrix degradation in osteoarthritis primes the superficial region of cartilage for mechanical damage. Acta Biomater. (2018) 78:320–8. 10.1016/j.actbio.2018.07.037 30059801

[47] Finnilä M Gupta SD Turunen M Hellberg I Turkiewicz A Lutz-Bueno V, et al. Mineral crystal thickness in calcified cartilage and subchondral bone in healthy and osteoarthritic human knees. J Bone Miner Res. (2022) 37:1700–10. 10.1002/jbmr.4642 35770824 PMC9540032

[48] Legrand J Marzin C Neogi T Norberciak L Budzik J Pascart T. Associations of changes in knee hyaline cartilage composition measured with dual-energy computed tomography in gout, aging and osteoarthritis. Cartilage. (2024) 15:283–92. 10.1177/19476035231172152 37312537 PMC11418446

[49] Linus A Tanska P Nippolainen E Tiitu V Töyras J Korhonen RK, et al. Site-specific elastic and viscoelastic biomechanical properties of healthy and osteoarthritic human knee joint articular cartilage. J Biomech. (2024) 169:112135. 10.1016/j.jbiomech.2024.112135 38744145

[50] Blumenkrantz G Stahl R Stahl R Carballido-Gamio J Zhao S Lu Y, et al. The feasibility of characterizing the spatial distribution of cartilage t(2) using texture analysis. Osteoarthritis Cartilage. (2008) 16(5):584–90. 10.1016/j.joca.2007.10.019 18337129 PMC2838772

